# Cultural Influences on the Regulation of Energy Intake and Obesity: A Qualitative Study Comparing Food Customs and Attitudes to Eating in Adults from France and the United States

**DOI:** 10.3390/nu13010063

**Published:** 2020-12-28

**Authors:** Maria Carlota Dao, Sophie Thiron, Ellen Messer, Camille Sergeant, Anne Sévigné, Camille Huart, Melinda Rossi, Ilyssa Silverman, Kylie Sakaida, Pierre Bel Lassen, Charlotte Sarrat, Laura Arciniegas, Sai Krupa Das, Nicolas Gausserès, Karine Clément, Susan B. Roberts

**Affiliations:** 1 Energy Metabolism Laboratory, Jean Mayer USDA Human Nutrition Research Center on Aging, Tufts University, 711 Washington Street, Boston, MA 02111, USA; anne.sevigne@etudiants.purpan.fr (A.S.); camille.huart1@gmail.com (C.H.); melinda.rossi.42@gmail.com (M.R.); isilverman415@gmail.com (I.S.); kyliesakaida@gmail.com (K.S.); Sai.Das@tufts.edu (S.K.D.); Susan.Roberts@tufts.edu (S.B.R.); 2 Laboratoire CERTOP, UMR CNRS 5044, Université Toulouse 2 Jean Jaurès, 31058 Toulouse, France; sophie.thiron@wanadoo.fr (S.T.); arciniegas.l.a@gmail.com (L.A.); 3 Friedman School of Nutrition Science and Policy, Tufts University, 150 Harrison Avenue, Boston, MA 02111, USA; Ellen.Messer@tufts.edu; 4 Assistance Publique Hôpitaux de Paris, Nutrition Department, CRNH Ile-de-France, Pitié-Salpêtrière Hospital, 83 boulevard de l’Hôpital, 75013 Paris, France; camille.sboy@yahoo.fr (C.S.); pierrebellassen@gmail.com (P.B.L.); karine.clement2@gmail.com (K.C.); 5 INSERM, Nutrition & Obesity—Systemic Approaches Research Group (NutriOmics), Sorbonne Université, 83 boulevard de l’Hôpital, 75013 Paris, France; 6 Danone Research, 128 Avenue de la Vauve, CEDEX, 91767 Palaiseau, France; Charlotte.SARRAT@danone.com (C.S.); Nicolas.GAUSSERES@danone.com (N.G.)

**Keywords:** food culture, obesity, weight management, eating behavior

## Abstract

(1) Background: The influence of food culture on eating behavior and obesity risk is poorly understood. (2) Methods: In this qualitative study, 25 adults in France with or without overweight/obesity participated in semi-structured interviews (*n* = 10) or focus groups (*n* = 15) to examine attitudes to food consumption and external pressures that influence eating behavior and weight management. Results were compared to an equivalent study conducted in the United States, thereby contrasting two countries with markedly different rates of obesity. Emerging key themes in the French data were identified through coding using a reflexive approach. (3) Results: The main themes identified were: (1) influence of commensality, social interactions, and pleasure from eating on eating behavior, (2) having a balanced and holistic approach to nutrition, (3) the role of environmental concerns in food consumption, (4) relationship with “natural” products (idealized) and food processing (demonized), (5) perceptions of weight status and management. Stress and difficulties in hunger cue discernment were viewed as important obstacles to weight management in both countries. External pressures were described as a major factor that explicitly influences food consumption in the U.S., while there was an implicit influence of external pressures through eating-related social interactions in France. In France, products considered “natural” where idealized and juxtaposed against processed and “industrial” products, whereas this was not a salient aspect in the U.S. (4) Conclusions: This first comparative qualitative study assessing aspects of food culture and eating behaviors across countries identifies both common and divergent attitudes to food and eating behavior. Further studies are needed to inform the development of effective behavioral interventions to address obesity in different populations.

## 1. Introduction

The prevalence of chronic diseases continues to increase globally [1,2,3,4], and obesity is a major underlying cause [5,6,7,8]. A wide variety of biological and psychosocial factors have been implicated in the development of obesity, with excess energy intake playing a dominant causal role [5,6,9,10,11,12,13,14,15,16]. However, the underlying causes of excess energy intake are multifactorial, and the role of food culture in obesity risk in different nations is poorly understood.

It is widely acknowledged that food intake is influenced by culture and varies geographically [17,18], as do attitudes toward eating for sociability, pleasure, environmental and farmer sustainability, and health [19]. Food culture can be defined as the attitudes, beliefs, and traditions about food and eating practiced by individuals, and the social relations and institutions connecting individuals around and through food. Both eating behaviors and food preferences may be influenced by prevailing food culture [20]. In turn, food culture may be determined or influenced by food availability, socioeconomic factors, societal and economic transitions, and information dissemination through various media [21]. It remains to be explored how food culture influences food consumption patterns within globalized food systems and diets, and how ideas of national diets, consumption of iconic foods associated with cultural belonging, and cultural values on unrestricted or restricted eating or eating styles, alone or in groups, influence intakes of different food groups and identity. While public health research on determinants of under- or over-eating examines how cultural food values influence a sense of well-being [19] or distress, particularly in relation to what are perceived deviations from “normal” body size or shape [22], a better understanding of the role of food culture in weight management is needed to support the development of effective interventions to address obesity globally.

France and the United States are high-income countries with considerable differences in food consumption patterns and eating behaviors, as previous research has shown [23,24,25,26,27,28,29,30]. These include differences in lifestyle and dietary patterns, socialization during meals, eating schedules, and attitudes to food and food production. Furthermore, there are different rates of obesity in these two countries, with the prevalence of obesity in U.S. adults being four times that of France [31]. The relationship between weight status and attitudes and perceptions about health and the food environment have not been comparatively assessed in both countries. Here, we implemented a qualitative assessment of attitudes and perceptions about food and weight management in adults of different weight status in France and compared them to a parallel study in the U.S. [32].

## 2. Materials and Methods

### 2.1. Study Recruitment

Participants were a convenience sample of adults residing in France (*n* = 25) with a body mass index (BMI) categorized as healthy (20 ≤ BMI < 25 kg/m^2^), overweight (25 ≤ BMI < 30 kg/m^2^), or obese (BMI > 30 kg/m^2^). Some were recruited during a research “open house” day at the Pitié-Salpêtrière Hospital, Nutrition Department in Paris, France. In addition, participants of ongoing studies were invited to take part in either the interviews or focus groups. This study was reviewed and approved by the Tufts University Health Sciences Institutional Review Board (Project #12820, approval date: 10 April 2018) and the Clinical Research Regulation Office at the Pitié-Salpêtrière Hospital (approval date: 1 July 2018). All study activities were conducted in compliance with the Global Data Protection Regulation. An information form describing the study and containing a statement about voluntary participation was distributed to all participants. Data collection was implemented in summer of 2018. Ten subjects completed individual interviews and 15 subjects participated in one of two focus groups. All research activities took place in meeting rooms in the Nutrition Department of Pitié-Salpêtrière Hospital. This group of participants was compared to a group of adults recruited in the U.S., whose results have been previously published [32]. Briefly, adults in the U.S. completed interviews (*n* = 10) or focus groups (*n* = 14) using the same methodology and script as described below. These participants had a BMI in the healthy range (*n* = 5 for interviews), or in the overweight/obese range (*n* = 5 for interviews, and *n* = 14 for focus groups). The analytical approach used in the U.S. study is comparable to the one presented here.

### 2.2. Interview and Focus Group Content

The scripts for the semi-structured interviews and focus groups were translated by native French speakers from the English version described previously [32], and consisted of questions developed by experts in nutrition, sociology, and anthropology, and by clinicians from the collaborating institutions. The two complementary qualitative study formats were used to study individual responses (interviews) as well as social dynamics and group reactions (focus groups) to the same questions.

Questions were developed within six topics related to eating behavior, culture, and attitudes to nutrition and health: (1) Attitudes to healthy/unhealthy food; (2) Drivers of food choices; (3) Attitudes toward body image, nutrition, and health; (4) Insularity, fatalism, and other generalized cultural traits; (5) Attitudes to hunger and restraint; and (6) External pressures to overeat or not eat. These categories were established based on the study team’s knowledge of factors associated with cultural background and weight management that could be considered in the creation of culturally relevant weight management programs.

Every interview and focus group began with an icebreaker exercise, where participants were asked about their opinions about foods in a food photo created at the Energy Metabolism Laboratory at Tufts University, available in [32]. The same interviewer (MCD) led nine interviews, and another team member (CSe) led one interview due to scheduling conflicts. A team member with a sociology background (ST) led the focus groups. Notetakers recorded non-verbal communication in all interactions.

### 2.3. Qualitative Data Analysis

Interviews and focus groups recordings were transcribed verbatim by native speakers. Quality checks for transcription errors were performed in randomly selected subsets of the transcripts and recordings. Transcripts were analyzed using Word and the NVivo^®^ software. The data collection approach was designed to be reflexive, to let interviewees describe how they positioned themselves with respect to food and dietary and nutritional expectations for eating behavior. The transcribed text was first sorted into the aforementioned question categories. Once the text was sorted, it was coded by two coders (a primary coder and a secondary coder), where key words and phrases were assigned code words that underwent multiple rounds of review by the study team. Initial codes were bundled into main codes. Disagreements on the coding process were resolved during weekly meetings and with the study coordinator. A code book including codes, definitions, and examples was created. The coded transcripts were read repeatedly, and subsequently categorized according to participant weight status, and again read multiple times. Main code frequency and transcript content led to the identification of salient themes for the entire study group, and by weight category. Concepts and themes based on the codes were identified through consensus of the study team [33,34,35].

## 3. Results

### 3.1. Characteristics of the French Participants

Table 1 summarizes participant characteristics by BMI category (healthy weight status vs. overweight or obese). Age ranges were similar between BMI categories. The self-reported average BMI was 22.0 ± 1.7 kg/m^2^ for the healthy weight group and 29.1 ± 3.6 kg/m^2^ for the overweight/obese group. The majority of participants was female, with 3 out of 25 male participants.

### 3.2. Food Preferences in Relation to Weight Status in French Participants

At the beginning of each interview, participants were asked to select their three favorite foods from a food photo created by the investigators [32]. The photo included foods that have been positively or negatively associated with chronic disease risk, as previously reported [32]. Participants in both BMI categories expressed feeling enjoyment from most foods on the photo, in some cases asking to select more than three choices, although one participant from each BMI category focused on dislikes rather than the foods they enjoy (Table 2). For example, a participant with healthy BMI said:

L1 (BMI status: normal): “Vous auriez dû me demander de barrer tous ceux que je ne mange jamais, ça aurait été plus facile.”

“You should have asked me to cross out what I don’t like, that would have been easier.”

A participant with high BMI had a similar opinion:

O4 (BMI status: obese): “…trois seulement? Il y a trop de bonnes choses en fait c’est ça.”

“…only three? There are too many good things, that’s the thing.”

The mean caloric content (kcal/serving) and calorie density (kcal/g) were calculated for the three foods selected from the photo during the interview icebreaker exercise. Averages for each BMI group were subsequently calculated. The average (SD) caloric content and density of the preferred foods was 377 (189) kcal/serving and 1.6 (1.20) kcal/g, respectively, in the group with healthy BMI and 353 (153) kcal/serving and 1.4 (0.5) kcal/g, respectively, in the group with high BMI.

### 3.3. Key Emerging Themes in Interviews and Focus Groups for French Participants

The key emerging themes from the French interviews and focus groups were: (1) influence of commensality, social interactions, and pleasure from eating on eating behavior, (2) having a balanced and holistic approach to nutrition, (3) the role of environmental concerns in food consumption, (4) relationship with “natural” products (idealized) and food processing (demonized), (5) perceptions of weight status and management. These themes are presented in detail in this section, and examples are provided both within the text and on Table 3.

#### 3.3.1. Commensality, Social Interactions, and Pleasure from Eating

For participants in France, the general attitude towards commensality or social eating was reflexive, with social interactions being instinctively considered significant determinants of eating behavior. For several participants, behaviors and emotions when eating alone differed from those experienced when eating in the company of others. The fear of judgement during social eating occasions was prevalent, but at the same time participants described their own judgement of the eating behavior of others. For example, a participant with obesity described her behavior when eating with colleagues as follows:

O4: “Si [ce] sont des évènements avec des collègues, pas mes proches collègues, pas les collègues avec qui je mange le midi qui me connaissent etc., mais un peu plus général je consomme très peu. Alors que si je suis avec des collègues que je vois régulièrement avec qui je suis très habituée, on sait chacun nos petits péchés mignons donc voilà on fait pas très attention à ce qu’on fait, on aura pas peur du regard.”

“If it is an event with colleagues, not my close colleagues, not the colleagues with whom I eat at midday who know me etc. but more generally, I will eat very little. However, if I am with my colleagues who I see regularly that I am very used to, we know each other’s quirks so we don’t pay a lot of attention to what we do, and we do not fear judgement.”

In addition, the importance of pleasure from eating was a prominent topic in both the interviews and focus groups, which is consistent with previous reports [27,30]. The eating context, such as eating alone versus eating with others, was linked to pleasure from eating. Furthermore, pleasure was deeply connected to the consumption of healthy foods produced in a way that was viewed as moral by the participant (e.g., locally sourced, or pesticide-free). For example, one subject described their views on wine consumption, as shown in Table 3.

Several participants in France expressed enjoying foods precisely due to their beneficial effects for health. For some participants, the enjoyment of healthier foods was sometimes acquired or learned:

L4: “Dans la société où l’on vit il y a beaucoup d’informations qui circulent sur la nutrition, donc on nous recommande de manger beaucoup de fruits et de légumes, pas trop salé, pas trop sucre, donc j’essaye de mettre ces principes en pratique. Du coup j’ai aussi développé un gout pour les fruits les légumes les céréales.”

“In the society in which we live there is a lot of information circulating on nutrition, and we are recommended to eat a lot of fruits and vegetables, not too salty, not too sweet, so I try to put these principles into practice. So I also developed a taste for fruits, vegetables and [whole grains] cereals.”

#### 3.3.2. Having a Balanced and Holistic Approach to Nutrition

Participants in France viewed nutrition holistically with health, general wellbeing, and morality. When considering the nutritional value of food, there was demonization of sugar, while certain foods considered healthier, such as dark chocolate, had the effect of lifting guilt upon consumption. When describing morality in food choices, the method of food production, origin of food, and rights of food producers were mentioned by most participants in both focus groups and interviews. Health is therefore integrated into a way of life, as expressed by a participant from one of the focus groups (Table 3).

In older participants, there was a general perception that this holistic view of nutrition is disintegrating in younger generations. Older participants had the impression that, due to the fast pace of current lifestyles, younger generations do not look after their own wellbeing and allot insufficient time to eating, which may be a result of growing constraints of daily life, such as having demanding work schedules:

L5 (older than 65 years): “Des gens de votre génération qui mangent dans la rue des trucs comme ça [junk, processed foods], ça je trouve ça vraiment dommage de ne pas pouvoir s’arrêter.”

“People of your generation who eat things like that [junk, processed foods] on the street, I find it really unfortunate not to be able to take a pause.”

#### 3.3.3. The Role of Environmental Concerns in Food Consumption

The most prominent theme identified in the data was a concern over food production and environmental factors that influence nutrition and health. There was frequent mention of factors that link health to food production and the environment, such as consumption of local foods, seasonality, pesticide use, food waste, concern over humane production of animal products such as red meat (however, red meat was an important food widely consumed by some participants), solidarity with farmers and food producers, traceability of food production, and pollution. Anxiety about pesticides in food were mentioned by all participants at least once during the focus groups and interviews. Some representative quotes from participants in France follow:

On seasonality:

L1: “J’ai pas encore acheté de tomates, parce qu’on n’est pas encore dans la saison des tomates.”

“I haven’t bought any tomatoes yet, because we’re not in the tomato season yet.”

On food production concerns:

O3: “Mes envies de découvertes sont plutôt freinées par justement des problématiques, de traçabilité, de ce que [la nourriture] contient sans que ce soit marqué, et quand ça vient des pays très loin, ça consomme en carburant, là aussi il faut ménager ...des choses bios qui arrivent de l’autre bout du monde. C’est pas non plus les même contraintes qu’ils ont eu que le bio en France. Et puis j’essaye de faire travailler le Français pour que les gens aient du travail.”

“My desire for discovery [of new food] is rather hampered precisely by traceability issues, what [food] contains without it being labeled, and when it comes from countries very far away it consumes fuel, there too we have to be careful... organic things that come from the other side of the world do not have the same regulations as organic foods in France.. And then I try to give work to the French so that people have jobs.”

Finally, a focus group participant expressed concern about pesticides in food, which were shared by the rest of the group (Table 3).

#### 3.3.4. Relationship with “Natural” Products (Idealized) and Food Processing (Demonized)

Nature and “natural” products were idealized by most participants in France and were considered superior to products with significant human intervention, processing, or industrialization. Foods that have been processed, or that are linked to industry, were considered detrimental to health and behavior (Table 3).

Another point of view was that food production should not be entirely under human control or standardized and should be left to natural processes. Generally, the countryside was considered healthy and beneficial for human health, and there was an appreciation for local products and traditional practices:

L1: “Les barres de céréales et ces machins-là [on food photo] [...] il vaut mieux manger un bon bout de fromage avec une barre de chocolat.”

“Cereal bars and these things [on food photo] [...] it is better to eat a good piece of cheese with a bar of chocolate.”

Focus group participant: “Les produits du terroir en général c’est du traditionnel et c’est souvent de la qualité”

“Local products in general are traditional and often of quality.”

Challenges were experienced by most participants with their food environment and the perceived cacophony of health and nutrition messages [26], characterized by excess of food advertisements, and dissemination of erroneous nutrition information and misleading or confusing health messages:

L1: “La publicité me dit ‘mange des chips, mange machin, bois du lait.’ Moi, Je ne tombe pas dedans.”

“The advertisements tell me “eat chips, eat this thing, drink milk.” I’m not falling for it.”

#### 3.3.5. Perceptions of Weight Status and Management

A variety of behaviors were described within the areas of weight management, body image, and hunger. Societal pressures and stigma with regard to weight status were perceived by a large proportion of participants, particularly those with overweight or obesity. In addition, there were different views about weight control. For example, one participant with healthy weight did not pay much attention to their weight:

L1 (BMI status: healthy): “…c’est seulement au changement de saison, là c’est l’été alors on met des robes et parce qu’il fait chaud. Si par exemple l’hiver arrive et que je mets un jean et que je vois que ça serre, je me dis que j’ai grossis. C’est que comme ça.”

“…only at the change of season, it is summer now so I put on dresses and because it’s hot. If for example winter arrives and I put on jeans and I see that they are tight, I tell myself that I have put on weight. That is just how it is.”

On the other hand, there were participants with high BMI who have been dieting since adolescence, and would not recommend these regimes to others (Table 3 and Table 4).

There was a clear distinction of opinions according to weight status, and these are described in the next section.

### 3.4. Attitudes and Behavior Descriptions in Relation to Weight Status and Management

As described in the methods section, interview and focus group answers were organized into topic categories. The following summaries compare answers between weight groups by topic. Even though BMI is an imperfect measure of weight and health status at the individual level [22], self-reported values were used to estimate whether individuals had a healthy or overweight/obese weight status. Representative quotes by BMI status are shown in Table 4.

#### 3.4.1. Drivers of Food Choices

Both BMI groups described drivers of food choice that included pleasure from eating, concern with food processing or pesticides in foods, and food price (Table 4). Seasonality was prominently mentioned in both individual and group settings and in both BMI categories. Health was an important driver of food choice for participants with healthy BMI and, to a lesser extent, to participants with high BMI. Conversely, availability, convenience, and taste were important drivers of food choice for the high BMI group and, to a lesser extent, for participants with healthy BMI. Some participants in the high BMI group expressed awareness of the lack of health benefits from certain foods they prefer. They also described eating at times because food was available or craved, and not practicing meal planning strategies.

#### 3.4.2. Attitudes Toward Body Image, Nutrition, and Health

Regardless of weight status, improving and maintaining health was the main reason to lose weight or maintain a healthy weight (Table 4). The participants with healthy BMI said they were not very concerned about their weight status, although maintaining a healthy weight was important for aesthetic reasons. Health was also an important factor for these participants, but more in relation to general wellbeing and the prevention of chronic diseases such as cancer, rather than obesity prevention. Factors influencing weight status included food production, eating practices during childhood, having a balanced diet, and sociability.

The participants with high BMI who had long-lasting issues with weight management talked about the challenges of dieting from an early age. Some had lost confidence in weight loss programs and regretted ever participating in them. Experiences included previous participation in multiple diet programs for rapid weight loss, weight fluctuations, and positive comments after weight loss from peers, family, and friends. For these participants, the main reasons to lose weight were to attain general wellbeing and to gain the approval of others. Emotions were described as significant drivers of overeating and weight gain. Furthermore, stress was described as an important obstacle to healthy eating and weight management. In some instances, nutritional advice from others was seen as stressful and overwhelming. Finally, perceived social pressures and discrimination in social settings or in the workplace due to overweight were viewed as major contributors to stress.

#### 3.4.3. Insularity, Fatalism and Other Generalized Cultural Traits

Responses to questions on food culture and eating practices during childhood and adulthood revealed that priority is given to traditional eating practices and local food consumption (Table 4). Despite giving such importance to local products, the terms “food culture” (“culture alimentaire” in French) and “nutritional heritage” (“patrimoine alimentaire” in French) were not commonly used by the participants in France. When asked to give their definition of these terms, participants generally referred to food culture as something that is transmitted from generation to generation (maternal influences having an important role here), while nutritional heritage refers to foods and eating practices that are representative of a country or a region. Openness to trying new foods and cooking techniques varied from person to person, but was not prevalent.

One participant in France who was originally from the Caribbean, used the term “food culture” describe their connection to their region of origin through food:

O4 (BMI status: obese): “Si je parle culture alimentaire, donc moi je viens des Antilles donc on aime les plats bien remplis, c’est jamais très light, c’est bien bourratif.”

“If I’m talking about food culture, I’m from the Caribbean, so we like dishes that are full, it’s never very light, it’s very filling.”

For several participants with high BMI, current eating practices were different from their childhood. For example, some participants commonly consumed fresh products and a balanced diet with portion control during childhood, but found these practices too restrictive in adulthood and so they currently described eating larger portion sizes. Conversely, other participants said they ate fewer vegetables and were required to eat all the food on their plates as children, and currently practiced the opposite behaviors. Finally, some participants with high BMI reiterated how difficult it is to manage problems of daily life while dealing with diets and weight management, and said that the traditional French eating schedule does not fit with their hectic lifestyles.

#### 3.4.4. Attitudes to Hunger and Restraint

Most participants in France said they usually finish everything on their plates, and mostly eat only when hungry, but they tend to snack when encountering something tempting. The main differences in craving behaviors between participants in different BMI groups were the types of food preferred, the quantity consumed, and the frequency of consumption through, for example, snacking. The participants with high BMI felt they had lost the ability to distinguish intrinsic hunger signals from cravings, while the participants with healthy BMI described effectively distinguishing these signals (Table 4).

The participants with healthy BMI, especially older participants, did not follow a strict eating schedule or rules about eating when not hungry. Some described listening to intrinsic signals, while others tended to eat enjoyable foods even when not hungry. Importantly, they described practicing strategies to avoid overeating. For example, even though they habitually finish everything on their plate, they plan portion sizes to avoid excessive consumption. This occurs both at home and away from home and may take different forms. For example, the serving size at home would be regulated while appetizers or dessert at the restaurant would be skipped.

Participants with high BMI described usually snacking when hungry, having a tendency to eat when food is available regardless of hunger (especially if the foods are considered palatable), and frequently eating until uncomfortably full. Eating was very dependent on emotions and was linked to eating compulsively and mindlessly. Some mentioned previously trying and often failing at different strategies to moderate food intake.

#### 3.4.5. External Pressures to Overeat or Not Eat

Participants tended to judge the eating behavior of others (Table 4). At the same time, some described changes in their eating behavior in front of others for fear of being judged. The participants with healthy BMI usually found food in excess at celebrations and little eating restriction by those present, which they tended to judge negatively. The main difference described between eating alone and eating with others is that eating alone allows one to focus more on the food than the social interaction. Feeling judged in social occasions was also seen in this group. For example, one participant experienced pressure from colleagues, who frequently questioned her food choices as a vegetarian.

For the high BMI group, food availability and social setting play a significant role in food consumption and eating behavior. They described difficulties in regulating food intake when too much food is available at celebrations, while eating alone is accompanied by guilt-inducing behaviors such as overeating. Similarly to the group with healthy BMI, they pay careful attention to the eating behavior of others.

### 3.5. Parallels and Contrasts of French Participants with a Comparable Study in the United States

A study conducted in the U.S. [32] used the same scripts, icebreaker exercise, and research design as in this study. Analysis of the U.S. interviews and focus groups led to the development of a model including both internal cues and external pressures, both perceived and overt, influencing eating behavior. Five domains of internal cues that influence eating behavior were identified: (1) hunger and satiety; (2) restraint; (3) disinhibition; (4) cravings; and (5) association of pleasure from eating and nutritional composition of food. In addition, five domains of external factors influencing food intake were identified: (1) environmental cues including food availability and variety; (2) normative expectations for dietary intake; (3) food palatability; (4) overt social pressures to overeat; and (5) perceived social expectations around eating. Of note, external overt and perceived pressures to overeat, especially in social settings, were identified as challenging by participants with high BMI in the U.S., while there was limited description of solutions to these challenges. Social pressures and perceived expectations from family members, friends and co-workers appeared to be especially problematic. This analysis led to a characterization of external factors that challenged healthy weight regulation, which could serve as targets for weight management intervention development.

Perceived external pressures to eat or not eat were present in both countries, but were slightly more prevalent and overt in the in the U.S. than in France. Furthermore, several participants in the U.S. mentioned weight management challenges when selecting foods on the photo while this topic was not mentioned in this section of the discussion by participants in France. However, participants in France mentioned stress and hectic lifestyles as obstacles to healthy eating. In addition, the discordance between pleasure and taste was not prevalent in France, while it was one of major themes identified in the U.S., consistent with previous reports [36,37].

The most notable difference between the two countries was seen in a greater concern over food production practices in France, particularly pesticide use, which was not mentioned as a priority for participants in the U.S. French participants also prioritized local food production and consumption, while in the U.S. local products or eating practices were not overwhelmingly preferred over foreign foods. In fact, they were considered unhealthy by some, including participants who were originally from the South of the U.S. Nevertheless, similar factors were also identified in relation to weight status, with health mentioned as a prominent driver of food choice by participants with healthy BMI, while taste was more commonly mentioned by participants with high BMI. Participants with healthy BMI described being more responsive to hunger and satiety cues than those with high BMI.

## 4. Discussion

This qualitative study explored the relationship between food culture, eating behavior, and attitudes to food consumption and weight management in adults with healthy or high weight status in France, and compared data to a parallel group of participants in the U.S. [32]. Salient themes from the interviews and focus groups in France were identified by the study team through analysis of the transcripts and code book, using a reflexive approach after categorizing answers according to sections of the semi-structured script. These themes were: (1) commensality, social interactions, and pleasure from eating, (2) having a balanced and holistic approach to nutrition, (3) the role of environmental concerns in food consumption, (4) relationship with “natural” products (idealized) and food processing (demonized), and (5) perceptions of weight status and management. Stigma related to overweight was prevalently expressed and associated with external pressures, concordantly with previous reports [38]. The group with overweight/obesity described challenging and long-lasting experiences with overweight and unsuccessful weight management, which were connected to stress and emotional eating.

When comparing the data from French and U.S. participants, stress and an inability to distinguish between hunger and craving cues were described as obstacles for weight management by participants with high BMI in both countries, while participants from the U.S. experienced greater external perceived and overt pressures to overeat and a strong discordance between the health and pleasure attributes of food. This is consistent with previous studies where the Intuitive Eating Scale-2 was administered, revealing an inverse association between overweight and intuitive eating (i.e., response to physiological hunger cues) in France [39] and the U.S. [40]. To our knowledge, this is the first qualitative comparison of eating behavior, including discernment of hunger and craving cues, and food culture with respect to weight status in France and the U.S. and calls for further investigation into culturally specific barriers to weight management.

The perceived synergy between healthfulness of food and palatability in the French participants, which was observed regardless of weight status, is consistent with previous results reported by Werle et al. for French adults [37]. This result was diametrically opposite to the discordance of taste and healthfulness in U.S. participants. In the U.S., there was also a tendency to categorize foods according to their nutritional attributes (i.e., healthy vs. unhealthy) with little room for nuance, leading to restriction, inhibition and guilt associated to “unhealthy”/ “tasty” foods [36,37]. This strict antagonistic categorization has not been commonly observed in France. These results suggests that weight status is an additional dimension for consideration in the relationship between healthfulness and palatability. Future studies should also include an assessment of how these views are evolving with time and whether there are generational differences. In support of this, a recent survey conducted in the U.S., France, Belgium, and the United Kingdom found a positive association between self-reported weight status and the tendency to believe that unhealthy food is tastier [41]. However, this tendency did not differ between countries, even though there was a lower prevalence of obesity in the French population. This discrepancy with our and previous [36,37] findings may be attributed to the different methodologies used. Interestingly, however, the authors commented on a globalization-driven convergence of attitudes to food in these countries, which also warrants further investigation.

While changing trends in food consumption were identified by participants in France, importance was given to traditional eating practices and food production methods. Participants expressed an idealized view of “natural” products, which were contrasted to the negatively viewed “industrial” or processed foods. Previous studies have shown that attention to food production and changes in consumption patterns have increased in French society [25]. This concern over food production methods was not as prevalent among participants from the U.S. Even though awareness and concern over the impact of food production on climate has been identified as a growing trend in subpopulations in the U.S. [42,43,44], it is less prevalent than in other regions in the world [44,45,46]. In connection with this topic, another difference identified in this study was that participants in France had a more holistic view of the role of food in health. Their relationship with food encompassed nutrition, general wellbeing, social interactions, and the environment. The identification of commensality and joy from eating as one of the salient themes in our data is consistent with previous characterizations of French eating culture with respect to the U.S. [47]. Morality was also seen as an integral part of food choice, and spanned consciously making healthy food choices, engaging in social eating, supporting local producers, and considering environmental sustainability.

Due to the small sample size and narrow age range, the findings from this study are not generalizable. Particularly the holistic language of balance and equilibrium, which closely follows official designated French gastronomic categories [48], suggests some highly educated and informed participants in France. Furthermore, sociodemographic information was not collected, which would have provided a richer context for the various views and opinions presented. The goal of each interaction was to cover all questions in the pre-specified topic categories. While this achieved a wide scope in the content of each interaction, sufficient depth regarding each individual question may have not been reached. An additional limitation is the categorization of these subjects according to self-reported BMI, which is a far from perfect approach to estimate health and weight status at the individual level. Future, larger studies are needed to assess the intersectionality of culture and weight status with regard to dietary patterns, meal frequencies and schedules, attitudes toward changing, more globalized eating behaviors, and perceptions of traditional eating practices in both countries.

Given that the sample of participants from racial or ethnic minorities was small and that this information was not systematically collected in both countries, a comparison of the meaning of “food culture” according to cultural heritage cannot be drawn here and would be the focus of future studies. In addition, future studies should also include collection of information on sociodemographic background (e.g., profession, educational level, household characteristics) and cultural background (e.g., place of birth, religion, cultural identity). General eating habits and behaviors should be systematically assessed. It is also important to consider the influence that the study setting and recruitment strategies may have had on the participant responses. Subjects knew they were being interviewed by nutrition experts and this may have biased some of the answers. Furthermore, certain recruitment strategies, such as the research “open-house day” may have attracted participants who have a higher than average interest in nutrition and health. Participants in both countries may have also been influenced by the research setting (i.e., hospital, nutrition research center). The study was conducted in two large cities (Paris and Boston), and so it included participants residing in urban or peri-urban environments who likely have had extensive exposure to health and nutrition information and perhaps more sensibility to certain topics such as pollution and food production. These participants may be more informed and give higher priority to nutrition than the general population.

## 5. Conclusions

Anthropologists, sociologists, gastronomists, and other food-studies specialists have explored propositions, “you are what you eat” or “tell me what you eat and I’ll tell you who you are” [49,50] to illuminate the influences of particular food cultures on national, sub-national, religious, or other ideological, values-based eating and nutritional outcomes [18]. Obesity is a serious public health concern worldwide that needs to be addressed in part through the development of culturally appropriate weight management recommendations. The development of such recommendations depends on an accurate and unbiased assessment of culturally specific barriers and opportunities to weight management. The findings from this study shed light on factors that need to be considered for the development of such strategies both in France and the U.S. Large scale assessments of food culture in relation to weight status employing mixed methods research are needed to enhance the effectiveness of weight management programs in different cultural contexts.

## Figures and Tables

**Table 1:** Characteristics of French participants.

	*N*	Mean (SD)	Min, Max
BMI < 25 kg/m^2^			
Gender (*N*): F/M	6/2		
Age (years)	8	61 (14)	38, 73
BMI (kg/m^2^)	8	22.0 (1.7)	19.6, 23.7
BMI ≥ 25 kg/m^2^			
Gender (*N*): F/M	16/1		
Age (years)	17	58 (13)	22, 74
BMI (kg/m^2^)	17	34.8 (5.1)	25.2, 44.8

F/M: Female/Male; BMI: body mass index.

**Table 2:** Food preferences, caloric content and caloric density from food photo exercise conducted during French interviews.

		Total Caloric Content for Selected Foods (Sum of kcal/Serving)		Energy Density for Selected Foods (Average kcal/g)	
ID	Food Selection	Individual	Average (SD)	Individual	Average (SD)
L1	Sardines/mackerel, spinach, bananas	326		1.9	1.6 (1.2)
L2	Spinach, broccoli	74		0.3	
L3	Baguette, eggs, strawberries, yogurt	467	377 (189)	1.1	
L4	V: Granola bars, pizza, bananas	562		3.4	
L5	Baguette, red wine, spinach	457		1.3	
O1	Broccoli, carrots, eggs, banana, spinach	227		1.0	1.4 (0.5)
O2	Nuts, apples, strawberries	364		2.1	
O3	Eggs, lentils, strawberries	238	353 (153)	1.1	
O4	Eggs, salmon (fish), pizza	606		1.8	
O5	Eggs, baguette, spinach	331		1.2	

Only interview participants (*n* = 10) completed this exercise. The energy density (kcal/g) and caloric content were calculated based on a typical serving size of each food (source: https://fdc.nal.usda.gov/). The total caloric density per individual was calculated by averaging caloric densities of the selected foods. The caloric content was calculated by adding the calories per serving for the selected foods. Averages and standard deviations (SD) were calculated by group.

**Table 3:** Key themes emerging from interviews and focus groups in France.

Key Theme (France)	Representative Quote
Commensality, social interactions, and pleasure from eating	L5 (BMI status: healthy): “Le vin, c’est un beau produit parce que c’est la conjonction de la terre, de la nature, du climat, du travail de l’homme, c’est quelque chose qu’on partage. Du vin seul c’est triste, je ne l’ai pas dit toute à l’heure mais du vin seul c’est triste. Je ne bois pas du vin seul, il faut partager les produits.” “Wine is a beautiful product because it represents the combination of the earth, of nature, of the climate, of the work of human beings, it is something that we share. Wine alone is sad, I did not say it earlier but wine alone is sad. I don’t drink wine alone, you have to share the products.”
Having a balanced and holistic approach to nutrition	Focus group participant: “Pour moi la santé c’est avoir un équilibre de vie [...]. Que ce soit à la fois sur le plan émotionnel, sur le plan physique, sur le plan un peu à tous les niveaux, être en harmonie.” “For me, health means leading a balanced life; to be in harmony emotionally, physically, and at all levels.”
The role of environmental concerns in food consumption	Focus group participant: “Notre situation je dirais est un peu grave maintenant. Parce qu’il y a 30-40 ans il n’y avait pas tous ces pesticides, il n’y avait pas, il suffisait de faire confiance pour manger.” “Our situation I would say is a little serious now. Because 30-40 years ago there weren’t all of these pesticides, there weren’t any. It was possible to trust what you ate.”
Relationship with “natural” products (idealized) and food processing (demonized)	L1 (BMI status: healthy): “Si vous allez dans un café ici à Paris, vous avez le café et vous avez le croissant mais jamais ça [donuts]. C’est vraiment le truc industriel que […] je ne mange pas.” “If you go to a cafe here in Paris, you have coffee and you have croissants but never that [donuts]. I really do not eat industrial [processed] junk foods.”
Perceptions of weight status and management	O2 (BMI status: obese): “…le conseil que j’ai donné à mes filles c’est de ne jamais faire de régime, jamais, je pense qu’à partir du moment où vous rentrez dans le système des régimes c’est une catastrophe.” “…my advice to my daughters is to never go on a diet, never, I think from the moment you get into the diet system it’s a disaster.” O2: « les régimes à mon avis m’ont détruite. » “Diets in my opinion have destroyed me.”

**Table 4:** Answer comparison by weight category in France.

Category	Healthy BMI Category	High BMI Category
Drivers of food choices	L4: « En fait, j’essaye de manger assez sain, dans la société ou l’on vie il y a beaucoup d’informations qui circulent sur la nutrition, donc on nous recommandé de manger beaucoup de fruits et de légumes, pas trop salé, pas trop sucré, donc j’essaye de mettre ces principes en pratiques. »	O5: « D’abord [I make choices] par rapport à ce qu’il me reste dans mon frigo, par rapport aussi à des envies ou des manques que j’ai. »
	“In fact, I try to eat quite healthy, in the society where we live there is a lot of information circulating on nutrition, so we are recommended to eat a lot of fruits and vegetables, not too salty, not too sweet, so I’m trying to put these principles into practice.”	“First of all, [I make choices] based on what I have left in my fridge, also based on desires or cravings that I have.”
Attitudes toward body image, nutrition, health	L2: « Des régimes, oui quand j’étais étudiante et des que je vois que je prends un ou deux kilos, mais je pense que je ne grossi pas si facilement que ça. »	O1: « Alors, la principale raison c’est que chaque fois que je vais voir un médecin pour quelque chose il me dit ça ne va pas, il faut perdre du poids. Maintenant il y a plus grave c’est devenu presque un handicap, par exemple dans mon lit j’ai du mal à me retourner… »
	“Diets, yes when I was a student and as soon as I see that I gain one or two kilos, but I think that I do not put on weight that easily.”	“So the main reason is that every time I go to the doctor for something he tells me, you have to lose weight. Now it is more serious, it has almost become a handicap, for example in my bed I can hardly turn around ... “ O2: « les régimes à mon avis m’ont détruite. » “Diets in my opinion have destroyed me.”
Insularity, fatalism, and other generalized cultural traits	L1: « Petite j’ai mangé correctement, quand ma mère faisait à manger on savait ce que c’était, on voyait que c’était une carotte, on voyait que c’était un poireau. »	O1: « Et les légumes pour eux [parents, grandmother] n’existaient que sous la forme du haricot vert ou des petits choux de Bruxelles, ou la salade. Tandis que maintenant non j’ai changé, complètement par rapport à eux et surtout sur les quantités car chez nous de cette époque-là comme chez beaucoup de gens il fallait finir tout le temps l’assiette, finir le plat, sinon c’était une injure à la cuisinière. »
	“As a child, I ate properly, when my mother cooked, we knew what it was, we saw that it was a carrot, we saw that it was a leek.”	“And vegetables for them [parents, grandmother] only existed in the form of green beans or small Brussels sprouts, or salad. While now I have changed, completely compared to them and especially on the quantities because for us at that time as with many people you had to finish the plate all the time, finish the dish, otherwise it was an insult to the cook.”
Attitudes to hunger and restraint	L1: « …si j’ai faim et que je suis chez moi, je vais manger, je m’en fiche de l’heure. Je vais écouter mon corps, oui. »	O2: « Ce qui ce passe c’est que quand on a des compulsions alimentaires, moi je sais que je n’ai pas faim, je sais que si je mange c’est pas bien, mais on ne peut pas s’en empêcher, c’est à dire que des fois on a même un système qui fait qu’on se rends compte qu’on a mangé qu’après avoir manger. Par exemple on va dans le placard, on prend ce qu’il y a, et après je suis en train de réfléchir, je ne me rends pas vraiment compte. »
	“…if I’m hungry and I’m at home, I’m going to eat, I don’t care what time. I will listen to my body, yes.”	“What happens is that when I eat compulsively, I know that I am not hungry, I know that if I eat it is not good, but I can’t stop myself. In other words, sometimes you have a system that makes you realize what you ate only after having eaten. For example, I go to the cupboard, I take whatever is there, and after I’m thinking, I didn’t even realize it.”
External pressures to overeat/not eat	L4: « …je trouve que le caractère convivial d’un repas joue [on eating behavior]. Je me concentre moins sur la nourriture au moment du repas, je me concentre plus sur l’interaction avec les autres, les discussions les... c’est un peu plus stimulant intellectuellement on va dire. »	O2: « En fait quand je mange toute seule je fais ce qu’il ne faut pas, je regarde internet, mon téléphone, je parle avec quelqu’un au téléphone. Donc oui c’est beaucoup plus difficile de manger toute seule. » “In fact when I eat alone I do the wrong thing, I look at the internet, my phone, I talk to someone on the phone. So yes it’s much more difficult to eat on your own.”
	“… I find that the sociability of a meal plays a role [on eating behavior]. I focus less on the food at mealtime, I focus more on interacting with others, the conversations... Let’s say it’s a little more intellectually stimulating.”	O1: “ Ah ben, maintenant oui, je regarde ceux qui vont vite, ceux qui ne vont pas vite, pour voir s’il y en a un qui va plus vite que moi. Donc j’ai constaté que ceux qui vont plus vite que moi ils étaient gros mais là la semaine dernière il y en avait une elle était maigre mais elle mangeait plus vite que moi. Donc oui je regarde, j’essaye de ne pas être la première à avoir terminé […] quand je vois la maigre là qui n’arrête pas de manger des frites ça m’énerve. Oui donc je fais attention à regarder ce que les autres mangent, voir s’ils en reprennent ou s’ils n’en reprennent pas. » “Ah well, now yes, I am looking at those who are [eating] fast, those who are not [eating] fast, to see if there is one who is [eating] faster than me. So I have found that those who [eat] faster than me are large, but last week there was one who was skinny, but she ate faster than me. So yes, I’m watching, I’m trying not to be the first to finish […] when I see the skinny girl who keeps eating fries it annoys me. Yes, so I’m careful to watch what others eat, to see if they go for seconds or not.”

## Data Availability

The qualitative data presented in this study are available on request from the corresponding author.
